# Sanitary Deficiences and Puerperal Diseases

**Published:** 1894-01-06

**Authors:** 


### CURRENT MEDICAL TOPICS.

### SANITARY DEFICIENCIES AND PUERPERAL DISEASE.

It is time that we arrived at some definite conclusions in regard to the origin of that group of diseases which are commonly classed together as puerperal fever, and which form by far the most fatal complication of labour.

Many men still believe that they are produced by the insanitary conditions in the midst of which so many women are confined, and some go so far as to think that exposure of the lying-in woman to the infection of the various specific infectious diseases, notably scarlet fever and erysipelas, will produce in her the malady recognised as puerperal fever; while others boldly declare that such things have nothing to do with it, that the condition is one of septicæmia due to the introduction from without of the organisms which produce decomposition and septic infection, and that in the great mass of cases they are introduced by the attendant. Nothing can be more positive than the position taken up by the believers in direct infection. They assert plainly that it is a dangerous doctrine to teach that puerperal fever can be caused by defective drains and sewer emanations, justifying the strength of this assertion by the further statement that anything which gives medical men a ready excuse for not adopting the use of antiseptics in their midwifery work, and offers a ready way of accounting for any case of puerperal disease which may occur in their practice, may rightly be stigmatised as "dangerous," from its direct effect in leading to laxity of practice. On the other hand, it seems perfectly certain that disease of some sort is produced in lying-in women, and produced in them in an exceptional degree, by exposure to emanations from foul sewers and defective drains. The testimony of many men of large experience, and of medical officers of health, cannot be ignored or put on one side, and it is to the effect that it by no means uncommonly happens that a disease classed as puerperal fever, or at all events a fatal fever among lying-in women, is found accompanied by insanitary conditions, and apparently caused by them.

The decision of this most important question is by no means so simple a matter as some people may think, nor is truth to be discovered by taking either one side or the other. The direct infectionists urge with great force that bad drains are so common that the discovery of the most glaring defect is but little proof that it is the cause of any accompanying disease; the sanitarians, on the other hand, point to the long generations of dirty hands by which women have through ages been accouched, and point to the survival of the race as proof that something else besides such a mode of infection is wanted to cause so fatal a disease; and, in fact, it is quite open to them to assert on their side that the doctrines preached by Drs. Cullingworth and Herman is dangerous, in so far as it leads people to be indifferent to their sanitary surroundings, trusting that all will be made safe by antiseptic precautions.

Perhaps the most useful light in elucidating this difficulty comes from analogy with other wounds, for, after all, the placental site, and the cervical and other lacerations are but wounds; and certainly, if we appeal to the experience of surgeons, we shall find much to support the proposition that sanitary conditions largely influence the progress of wounds; at the same time, fortunately, we shall find also that this evil influence can be prevented by antiseptic precautions. Here, then, is in all probability the key to safe treatment: Even if bad drains set up puerperal septicæmia, they do so *viâ* the discharges, and the infection can be prevented by antiseptic methods. We are all agreed, absolutely and without doubt, that the infection of a deep wound with septic micro-organisms will lead to septic poisoning, and often to general septic infection; and the experience of surgeons during the last twenty years has shown clearly, and with hardly the possibility of error, that such an infection may take place from the hands or instruments of the operator. So far analogy tells us only what we know from experience, that puerperal septicæmia may be carried from labour to labour as pyæmia and phagedæna used to be from operation to operation.

But are we to say that because fingers can carry infection it can arrive in no other way? This is the very essence of the question, and again analogy with other forms of wounds gives us the answer.

No one can doubt, looking to old pre-antiseptic days, and even to present experience where antiseptic precautions are not used, that the condition of the house has much to do with the progress of accidents and operations under its roof. The large decomposition of permanganates on testing sewer-poisoned air points to large organic impurity; the tendency of such air to cause decomposition, tainting meat, &c., points to this organic impurity being organised, being alive; and its tendency to taint the discharges of wounds, and thus to render them septic, unless protected by antiseptic means, is but another illustration of the same fact. It was, and it is, a matter of common experience that wounds treated by ordinary water dressings are influenced in their progress by the condition of the air around. Why, then, should the great wound caused by child-birth, with its prolonged discharges, be invulnerable to the same evil influences? We have, indeed, no reason to believe that it is. Just as the meat in a drain-poisoned larder goes bad, so do the discharges of the lying-in woman, and from them she becomes infected. There is no necessity to raise the hypothesis that the tissues of a woman living in a poisonous atmosphere become a favourable breeding ground for septic germs. Even the rudest health is no bar to septic poisoning, and the strongest woman may suffer from septicæmia if the discharges bathing her internal wounds become septic. The whole question then is, must this septic infection be introduced by doctors or nurses' hands, or may it not creep up from external discharges just as it creeps up a psoas abscess which is allowed to burst into a poultice? We can have no possible doubt that it may occur either way, and that, therefore, it may be influenced by defective sanitation just in the same way as the psoas abscess would be.

Considerations of this kind make it clear that we cannot go with those who say that the occurrence of puerperal septicæmia is uninfluenced by the sanitary condition of the house; at the same time they make it extremely probable that, even if the drains are in fault, the disease arrives *viâ* the discharges, as in other wounds, and can be prevented by antiseptic precautions. We have no right to separate the great surgical operation performed by nature in the act of parturition from other surgical procedures, the same principles must govern all, and whether infection threatens from fingers or from drains, antiseptic methods ought to keep it at bay. Some of the greatest triumphs of antiseptic surgery were in early days, when the cases were surrounded by infection and yet did well, and there is but little doubt that carefully-planned antiseptic methods are equally capable of warding off puerperal septicæmia, even though the drains be rotten and the sewers foul; as indeed is shown by a large and yearly growing experience.

At the same time it must be remembered that lying-in women may suffer from drain poisoning, probably even in greater degree than others, and we see no reason for refusing to accept the evidence of those who have observed instances of such disease, and have noted how rapidly they have recovered when removed from their bad surroundings. No doubt there are some, nay many, such cases, but drain poisoning is not puerperal septicæmia, nor is this latter likely to be cured by change of room.

